# Association between Single Nucleotide Polymorphisms in Cardiovascular Developmental Critical Genes and Hypertension: A Propensity Score Matching Analysis

**DOI:** 10.1155/2020/9185697

**Published:** 2020-03-19

**Authors:** Zhiqiang Zhao, Chunmei Gong, Yanfang Gao, Xiaoli Liu, Sai Wu, Heping Zhao, Xiaohui Wang, Huaixiang Liu, Chen Xiao, Jieyi Liu, Jiong Li, Yun He

**Affiliations:** ^1^Department of Preventive Medicine, School of Public Health, Sun Yat-sen University, Guangzhou 510080, China; ^2^Labortory Research Institution, Shenzhen Center for Chronic Disease Control, Shenzhen 518020, China; ^3^Key Laboratory of Prevention and Treatment of Cardiovascular and Cerebrovascular Diseases, Ministry of Education, Gannan Medical University, Ganzhou 341000, China; ^4^Department of Clinical Epidemiology, Aarhus University Hospital, Aarhus 8200, Denmark

## Abstract

Cardiovascular development critical genes are key determinants in cardiovascular diseases. We hypothesize that SNPs in these genes may play critical roles in the development of hypertension. Therefore, we enrolled 516 paired hypertension patients and controls in a total of 2,742 subjects in a cross-sectional population study by the propensity score matching (PSM) method. Twenty-one SNPs from 5 cardiovascular developmental related genes were detected by the improved multiplex ligase detection reaction (iMLDR) method. Conditioned logistic regression under three different genetic models, namely, additive model, dominant model, and recessive model, was performed. The odds ratio (ORs) and 95% confidence intervals (95% CIs) were used to estimate the associations of SNPs with hypertension. We found that the distribution of genotypes at rs833061, rs3025010, and rs699947 within the VEGFA gene and the distribution of alleles at rs3025010 in hypertension subjects were different from those in controls. Both rs833061 and rs3025010 were associated with hypertension in crude models, but only rs3025010 remains associated with hypertension after adjusting with confounding factors in the additive model and the dominant model. We also found that hypertension subjects with C/T and C/C genotypes at rs3025010 had lower SBP and DBP levels. In addition, rs3025010 could interact with rs6784267 within the CCM3 gene in the association. In conclusion, our findings suggest that rs3025010 may play a role in the pathogenesis of hypertension, which may be a potential target for individualized prevention and treatment of hypertension.

## 1. Introduction

Hypertension is a common chronic disease and a major risk factor for stroke, myocardial infarction, and kidney failure [1, 2]. Single nucleotide polymorphisms (SNPs) are DNA sequence polymorphisms caused by a single nucleotide variation at the genome level and are the most common human genetic variants [3]. Genome-wide association studies (GWASs) have enabled the discovery of common genetic variation contributing to normal and pathological traits [4, 5], suggesting that the use of SNPs as biomarkers is useful for the screening of susceptible populations and is conducive to genetic diagnosis, gene therapy for hypertension [6, 7].

It is important to note that cardiovascular development critical genes are key determinants in cardiovascular diseases [8, 9]. Animal model studies showed that the deficiency of them is lethal. Taken CCM3 as an example, complete CCM3 knockout mice died early, whereas heterozygous deletion could survive and grow like wild-type, which suggests that it may act as a hidden primer for cardiovascular diseases in adulthood [10, 11]. However, there have been only limited significant loci of these genes which were found in GWASs [12]. Interestingly, we recently demonstrated that CCM3 gene polymorphism was associated with elevated blood pressure based on an arsenic-exposed population [13]. Therefore, we hypothesize that SNPs in cardiovascular developmental genes may play critical roles in the development of hypertension.

Propensity score matching (PSM) is a commonly used research method to solve the confounding bias in observational investigations [14]. This method was firstly proposed by Paul Rosenbaum and Donald Rubin in 1983 [15]. In recent years, it has been widely used in observational studies of nonrandomized large samples [16–18]. PSM method displays power improvement in identifying causal SNPs over other state-of-the-art methods. We employed PSM method and conditional logistic regression analysis in a population-based cross-sectional study to identify whether the putative functional SNPs are associated with hypertension in cardiovascular developmental critical genes.

## 2. Methods

### 2.1. Populations

This study was approved by the Medical Ethics Committee of the School of Public Health, Sun Yat-sen University. The subjects in this study were adults who had physical examination in a physical examination center in Shenzhen city from July 2013 to January 2014. They were mainly employees of government departments, enterprises, and institutions. The individual data of the subjects, including age, gender, BMI, hypertension family history (more than one member of the immediate family has hypertension), smoking (current smoking or the cumulative smoking time ≥6 months), drinking (alcohol consumption ≥3 g/day), salt intake, and physical activity (moderate or heavy intensity activities lasting more than 10 minutes in work and household activities, ≥1 day/week) were collected by specially trained investigators through questionnaires. The clinical examination of blood pressure, ultrasound, and biochemical parameters was measured by professional medical doctors and nurses. Subjects with severe anemia, severe heart and kidney insufficiency, urinary tract infection, nephritis, endocrine disorders, and pregnant women were not included. All subjects were divided into hypertension and control groups according to blood pressure levels and clinical information. All individuals signed informed consent.

**Figure 1:**
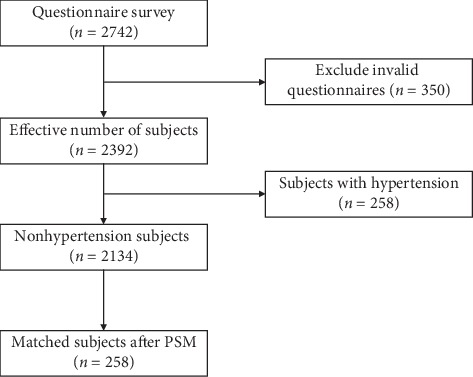
The flow chart of study population selection.

After questionnaire quality screening, clinical examination, medical history consultation, medical history review, and doctor's judgment, 2,392 effective subjects were selected. Among them, 258 subjects with hypertension were finally included in this study, and 258 healthy controls were matched by the propensity score method in 1 : 1 manner. A flow chart of the design of this study is illustrated in Figure 1.

### 2.2. Blood Pressure Measurement

Blood pressure was measured by standard procedures. The classification and definition of blood pressure were referred to the WHO/ISH guidelines 2003. Systolic blood pressure ≥140 mmHg and (or) diastolic blood pressure ≥90 mmHg was defined as hypertension. In addition, individuals who reported taking antihypertensive drugs were also considered as hypertension patients. The blood pressure level to 120–139/80–89 mmHg was set as a normal high value, with systolic pressure <120 mmHg and diastolic pressure <80 mmHg as ideal blood pressure. Subjects with both normal high value and ideal blood pressure were defined as normal.

### 2.3. Propensity Score Matching (PSM)

The covariates used for the calculation of the propensity score were age, sex, BMI, waist circumference, smoking history, drinking history, physical activity level, salt intake, total cholesterol, triglycerides, and blood glucose. Subjects with hypertension and healthy controls were matched at a ratio of 1 : 1 by the propensity score. In brief, open PSM module in SPSS first, then select nearest neighbour matching, enter ID variable as the serial number, enter binary treatment indicator (0 = control and 1 = hypertension), enter covariates for scoring, and finally match individuals exactly based on selected variables. The matching efficiency was assessed by outputs such as relative multivariate imbalance L1 metric and propensity score distribution histograms. The L1 metric is theoretically between 0 and 1. The smaller the L1 metric, the better the matching result. And if the distribution histograms between the postmatching group and the control group is similar, it indicates that matching is good [18, 19]. Given the association with hypertension in the logistic work model, we postulated that quantitative blood pressure levels would be lower in individuals with the minor allele at expected SNPs. Thus, we further studied the effect of SNPs on blood pressure traits in hypertension patients. For these subjects, the PS was matched in a ratio of 1 : 1 : 1 among the three genotypes by hand.

### 2.4. Selection and Genotyping of SNPs

DNA extraction was referred to the blood genomic DNA extraction kit instructions (Tiangen Biotech, Beijing). The tag SNPs were verified in HapMap Project and selected by using Haploview software (version 4.2) with the following filters: minor allele frequency (MAF) > 0.1 and *r*^2^ > 0.8. Finally, there were totally 21 SNPs selected, which include 3 sites (rs9818496, rs3804610, and rs6784267) from CCM3, 3 sites (rs55805015, rs2277538, and rs3212278) from DLL4, 4 sites (rs7667298, rs2305948, rs13109660, and rs7671745) from KDR, 2 sites (rs3124591 and rs73668310) from Notch1, and 9 sites (rs833061, rs10434, rs833069, rs3025010, rs3025053, rs699947, rs2146323, rs3025035, and rs3025030) from VEGFA. The specific information of these SNPs is listed in Supplemental [Supplementary-material supplementary-material-1]. Genotyping was done by the improved multiplex ligase detection reaction (iMLDR) method (Genesky Biotech, Shanghai) as previously described [20].

### 2.5. Statistical Analysis

Quantitative data were expressed as mean ± SD (standard deviation), which were compared using the *t*-test or one-way ANOVA using the *χ*2 test. The difference in the distribution of genotype frequencies between the two groups was tested using *χ*2 test. Conditioned logistic regression analysis was conducted to investigate the association of the differentially distributed SNPs with hypertension under three different genetic models including additive model, dominant model, and recessive model. In conditioned logistic regressions, the odds ratio (ORs) and 95% confidence intervals (95% CIs) were used to estimate the associations of SNPs with risk of hypertension. We also adjusted for SNPs that changed the matched regression coefficients by at least 10 percent. General linear regression was also performed to investigate the potential interaction among SNPs on blood pressure levels. The process of PSM was conducted by the PSM module in SPSS, and all statistical analyses were performed by SPSS version 20.0 software. *P* < 0.05 was considered as the statistically significant level.

## 3. Results

### 3.1. Basic Characteristics

A total of 2,742 questionnaires were issued upon the physical examination, and 2,392 samples including complete questionnaires and physical examination data were collected (response rate, 87%). Among them, there were 258 subjects of hypertension and 2,134 subjects of normal blood pressure. The hypertension group and the control group (including normal high values and normal blood pressure) were 1 : 1 matched, and a total of 516 subjects were enrolled in this study. The matched L1 statistic is 0.050, less than 0.522 (value before matching), suggesting that the match was good (Supplemental [Supplementary-material supplementary-material-1]). After matching, the distribution of hypertension and control groups was similar, further suggesting a good validity of matching (Supplemental [Supplementary-material supplementary-material-1]). Besides, we estimated the test power of our sample size when *α* level equals 0.05 and OR equals 1.5, and the results indicated that our study could provide 58.99% and 88.79% power for detection of genetic variation with a MAF of 0.1 and 0.5, respectively (Supplemental [Supplementary-material supplementary-material-1]). Systolic blood pressure, diastolic blood pressure, and pulse pressure in the hypertension group were significantly higher than those in the control group (Table 1).

### 3.2. The Distribution of Genotypes and Alleles

Among the polymorphism sites submitted, 19 SNP sites were analyzed since there was only 1 type of genotype in all subjects from rs73668310 (Notch1) and rs55805015 (DLL4) sites. After removing unreliable samples in the genotyping step, we finally obtained 225 pairs of good quality genotyping data. The quality control samples had the same reproducibility, and the negative controls had no band. All SNPs' response rates were greater than 98%, and the minimum alleles were consistent with HapMap-HCB data (Supplemental [Supplementary-material supplementary-material-1]). The results showed that the distribution of rs833061, rs3025010, and rs699947 genotypes in the VEGFA gene were significantly different between the hypertension group and the control group (*P* < 0.05) (Table 2). The allele frequency distribution also showed that there were significant differences in rs3025010 loci (*P* < 0.05) (Table 3).

### 3.3. The Association between SNPs and Hypertension

Because rs833061 and rs699947 were linearly correlated, we only studied rs833061. In additive model, the univariate analysis results showed that rs833061 and rs3025010 were associated with hypertension (*P* < 0.05), but only rs3025010 remained associated with hypertension in multivariate analysis after adjusting with confounding factors (OR = 0.103, 95% CI: 0.019–0.551, *P*=0.008). In the dominant model, the univariate analysis results showed that neither rs833061 nor rs3025010 was associated with hypertension (*P* > 0.05), but rs3025010 became associated with hypertension in multivariate analysis after adjusting with confounding factors (OR = 0.354, 95% CI: 0.147–0.854, *P*=0.021). In the recessive model, although rs833061 and rs3025010 were associated with hypertension, neither of the associations were significant in multivariate analysis after adjusting with confounding factors (*P* > 0.05). These results suggest that rs3025010 (CC genotype) may have an independent effect on hypertension (Table 4).

### 3.4. The Effect of SNPs on Blood Pressure Levels

**Figure 2:**
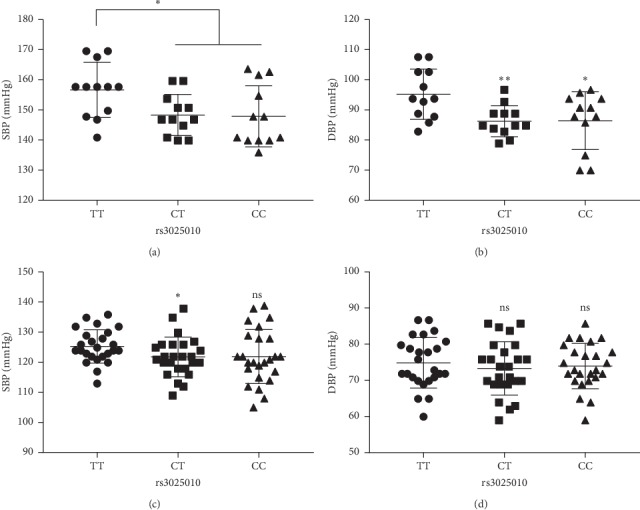
Minor allele at rs3025010 decreases blood pressure levels in hypertension subjects (a, b) and controls (c, d). SBP: systolic blood pressure; DBP: diastolic blood pressure. *N* = 12 for each genotype group in hypertension subjects. *N* = 25 for each genotype group in control subjects. ^*∗*^: *P* < 0.05 and ^*∗∗*^: *P* < 0.01.

In hypertension subjects with no hypertensive treatment, a total of 36 matched subjects were obtained, with 12 samples of each genotype at rs3025010 (TT, CT, and CC). We found subjects with CT and CC genotypes at rs3025010 had lower SBP and DBP levels although there were significant differences when compared with the T/T genotype (Figures 2(a) and 2(b)). In control subjects, a total of 75 matched subjects were obtained, with 25 samples of each genotype at rs3025010 (TT, CT, and CC). We found the levels of SBP and DBP were slightly lower in subjects with CT and CC genotypes at rs3025010, and only the difference of SBP was significant for the CT genotype when compared with the TT genotype (Figures 2(c) and 2(d)).

### 3.5. The Interactive Effect between SNPs in the CCM3 Gene and rs3025010 on Hypertension

Although the distribution of genotypes and alleles in SNPs from the CCM3 gene was not different between hypertension and control groups, our previous study found that CCM3 gene polymorphism is associated with hypertension in an occupational population exposed to low-level arsenic, and SNPs in the CCM3 gene could interact with it, suggesting that the CCM3 gene may assist with other factors. Therefore, we further examined the interaction between rs3025010 and SNPs of CCM3 genes. Among three SNPs in CCM3, we found that rs6784267 interacts with rs3025010 in the additive model and the dominant model but not in the recessive model. In particular, rs3025010 (CC) interacted with rs6784267 (C/T) in the additive model, and rs3025010 (C/T + C/C) interacted with rs6784267 (C/T + T/T) in the dominant model even after adjusting with confounding factors (Table 5).

## 4. Discussion

In this study, we found that rs3025010 within the VEGFA gene was associated with hypertension, and hypertension subjects with C/T and C/C genotypes at rs3025010 had lower SBP and DBP levels. In addition, rs3025010 could interact with rs6784267 within the CCM3 gene and further affecting hypertension.

Identifying putative functional SNPs associated with a disease can provide targets for gene therapy as early as possible, opening therapeutic strategies for precision and preventive medicine [21, 22]. Whole genome sequencing and screening for susceptible populations reported a suggestive association of SNPs and hypertension [23–25]. In addition, emerging technologies have been developed to repair DNA by editing the wrong bases [26, 27]. However, less is known about putative functional SNPs in the critical genes that are associated with hypertension. According to our preliminary work and literature review, cardiovascular development critical genes such as CCM3, DLL4, KDR, Notch1, and VEGFA contain multiple SNP sites, and bioinformatics data suggest that certain SNP sites may affect protein expression and function. In this study, with the assistance of the PSM method to adjusting certain definite risk factors for hypertension, we found that three SNPs in the VEGFA gene named rs833061, rs3025010, and rs699947 were differential-distributed in hypertension, indicating that these three SNPs could be potential independent risk factors in hypertension.

VEGFA regulates vascular development during embryogenesis and organ formation under physiological condition [28]. Disruption of this gene in mice resulted in abnormal embryonic blood vessel formation. This gene is upregulated in many known tumors, and its expression is correlated with tumor stage and progression [29, 30]. A recent study showed that polymorphisms in the VEGFA gene may affect the antihypertensive responses to enalapril [31]. In particular, A/A genotype at the rs699947 site is associated with a more intense decrease in blood pressure in response to enalapril, underlying its protective effect in hypertension, consistent with our results observed among rs699947, rs833061, and rs3025010. Chen et al. found that rs3025010 is possibly associated with a reduced risk of human brain arteriovenous malformation [32]. Interestingly, in our study, we found that rs3025010 was negatively associated with hypertension, and C/T and C/C genotypes which contain minor allele had lower SBP and DBP levels. This consistency suggests that the rs3025010 variant may play similar roles in cardiovascular diseases. To the best of our knowledge, this study is the first direct report of the association of VEGFA gene polymorphism at rs3025010 with hypertension.

The vast majority of GWAS tag SNPs lie in intergenic or intronic regions (approximately 88%) and therefore are likely to influence gene regulations [33]. rs3025010 is located in the fifth intron. Based on searching results from ENCODE (data not shown), we found that there were some candidate cis-regulatory elements (ccREs) near this locus including H3K27ac, which is an enhancer marker, suggesting that this locus region has the function of regulating target gene expression. Our study shows that the polymorphism of this locus is associated with hypertension, and the blood pressure level varies among different genotypes, suggesting that the polymorphism of this locus may affect the expression of genes related to blood pressure regulation through regulating the activity of enhancers or binding transcription factors.

We consider that SNPs in CCM3 and other genes may affect blood pressure by interacting with the VEGFA gene. Furthermore, our previous study found that the CCM3 gene may represent a novel susceptibility gene for hypertension in a population with arsenic exposure [13]. Therefore, we step forward to analyze the SNP-SNP interactions in this study. As expected, we found rs6784267 in the CCM3 gene interacted with rs3025010 in the VEGFA gene while both of our studies did not found associations between CCM3 gene polymorphism and hypertension, which is in line with our previous hypothesis that the CCM3 gene could affect blood pressure in a manner of interacting with other factors.

One strength of our study is that we used propensity score matching to adjust many covariates to produce unbiased estimates of the treatment effects [14]. Propensity score matching can also be very cost-effective [34]. Besides, although the sample size was relatively small, we could provide 58.99%∼88.79% power for detection of genetic variation for the selected tag SNPs. Our study also has limitations since we mainly examined the association between SNPs with hypertension. SNP site in the genomic DNA loci will affect the expression and function of the target gene and then further lead to cardiovascular dysfunction. Thus, further study is needed to explore its function and molecular mechanisms.

In summary, our findings suggest that rs3025010 in VEGFA is associated with hypertension. We also observed lower levels of SBP and DBP for C/T and C/C genotypes at rs3025010 in hypertensive subjects. In addition, rs3025010 could interact with rs6784267 within the CCM3 gene to affect the risk of hypertension. In depth studies are warranted to determine the target gene and investigate its potential role in the development of hypertension. This may improve our understanding of the role of cardiovascular development-related genes in the maintenance of blood pressure homeostasis. These findings will shed new light on gene diagnosis, gene therapy, and individualized therapy in hypertension. The results and discussion may be presented separately, or in one combined section, and may optionally be divided into headed subsections.

## Figures and Tables

**Table 1:** The baseline characteristics between hypertension and control groups.

Categories	Control	Hypertension	*P* value
Age (years)	45.33 ± 11.80	46.34 ± 13.57	0.36
Gender			0.22^*∗*^
Male, *N* (%)	180 (69.77)	166 (64.34)	
Female, *N* (%)	78 (30.33)	92 (35.64)	
Family history			0.21^*∗*^
No, *N* (%)	114 (44.19)	99 (38.37)	
Yes, *N* (%)	144 (55.81)	159 (61.63)	
BMI (kg/m^2^)	25.37 ± 3.62	25.48 ± 3.1	0.72
Salt (g/month)	258.79 ± 173.39	265.83 ± 229.84	0.30
Smoking history			0.99^*∗*^
No, *N* (%)	223 (86.43)	223 (86.43)	
Yes, *N* (%)	35 (13.57)	35 (13.57)	
Drinking history			0.72^*∗*^
No, *N* (%)	142 (55.04)	147 (56.98)	
Yes, *N* (%)	116 (44.96)	111 (43.02)	
Physical activity			0.75^*∗*^
No, *N* (%)	55 (21.32)	59 (22.87)	
Yes, *N* (%)	203 (78.68)	199 (77.13)	
TC (mmol/L)	5.46 ± 1.01	5.48 ± 1.03	0.76
TG (mmol/L)	2.37 ± 2.63	2.26 ± 2.22	0.60
Glucose (mmol/L)	5.69 ± 1.02	5.84 ± 1.24	0.12
SBP (mmHg)	121 ± 10	148 ± 12	<0.01
DBP (mmHg)	74 ± 8	88 ± 9	<0.01
Pulse pressure (mmHg)	47 ± 9	60 ± 14	<0.01

^*∗*^ *χ* ^2^ test. Bold font indicates *P* values less than 0.05. BMI, body mass index; TC, total cholesterol; TG, triglyceride; SBP, systolic blood pressure; DBP, diastolic blood pressure.

**Table 2:** The distribution of SNP genotypes between the control and hypertension groups.

Variables	Control			Hypertension			*P* value
	Ref. homo	Hetero	Alt. homo	Ref. homo	Hetero	Alt. homo	
rs2277538 (DLL4)	189 (84.0)	34 (15.1)	2 (0.9)	183 (81.3)	40 (17.8)	2 (0.9)	0.75
rs3212278 (DLL4)	94 (41.8)	115 (51.1)	16 (7.1)	113 (50.2)	91 (40.4)	21 (9.3)	0.07
rs7667298 (KDR)	90 (40.0)	98 (43.6)	37 (16.4)	88 (39.1)	113 (50.2)	24 (10.7)	0.15
rs2305948 (KDR)	165 (73.3)	59 (26.2)	1 (0.4)	159 (70.7)	63 (28.0)	3 (1.3)	0.54
rs13109660 (KDR)	103 (45.8)	97 (43.1)	25 (11.1)	105 (46.7)	100 (44.4)	20 (8.9)	0.73
rs7671745 (KDR)	98 (43.6)	98 (43.6)	29 (12.9)	99 (44.0)	93 (41.3)	33 (14.7)	0.82
rs3124591 (NOTCH1)	201 (89.3)	24 (10.7)	0 (0.0)	200 (88.9)	25 (11.1)	0 (0.0)	0.88
rs9818496 (PDCD10)	167 (74.2)	57 (25.3)	1 (0.4)	170 (75.6)	54 (24.0)	1 (0.4)	0.95
rs3804610 (PDCD10)	168 (74.7)	56 (24.9)	1 (0.4)	172 (76.4)	52 (23.1)	1 (0.4)	0.91
rs6784267 (PDCD10)	90 (40.0)	106 (47.1)	29 (12.9)	95 (42.2)	96 (42.7)	34 (15.1)	0.60
rs833061 (VEGFA)	127 (56.4)	73 (32.4)	25 (11.1)	133 (59.1)	81 (36.0)	11 (4.9)	0.04^*∗*^
rs10434 (VEGFA)	142 (63.1)	77 (34.2)	6 (2.7)	133 (59.1)	79 (35.1)	13 (5.8)	0.24
rs833069 (VEGFA)	86 (38.2)	105 (46.7)	34 (15.1)	72 (32.0)	108 (48.0)	45 (20.0)	0.25
rs3025010 (VEGFA)	117 (52.0)	83 (36.9)	25 (11.1)	134 (59.6)	79 (35.1)	12 (5.3)	0.03^*∗*^
rs3025053 (VEGFA)	168 (74.4)	53 (23.6)	4 (1.8)	161 (71.6)	59 (26.2)	5 (2.2)	0.75
rs699947 (VEGFA)	127 (56.4)	73 (32.4)	25 (11.1)	153 (59.1)	93 (36.0)	12 (4.9)	0.04^*∗*^
rs2146323 (VEGFA)	133 (59.1)	71 (31.6)	21 (9.3)	139 (61.8)	73 (32.4)	13 (5.8)	0.36
rs3025035 (VEGFA)	158 (70.2)	60 (26.7)	7 (3.1)	155 (68.9)	63 (28.0)	7 (3.1)	0.95
rs3025030 (VEGFA)	152 (67.6)	69 (30.7)	4 (1.8)	143 (63.6)	78 (34.7)	4 (1.8)	0.66

Ref. homo: reference homogeneous major allele genotype; hetero: heterogeneous genotype; alt. homo: altered homogeneous minor allele genotype. ^*∗*^*P* values have significance.

**Table 3:** The distribution of SNP allele frequencies in control and hypertension groups.

Variables	Control		Hypertension		*P* value
	Ref.	Alt.	Ref.	Alt.	
rs2277538 (DLL4)	412 (91.6)	38 (8.4)	406 (90.2)	44 (9.8)	0.49
rs3212278 (DLL4)	303 (67.3)	147 (32.7)	317 (70.4)	133 (29.6)	0.31
rs7667298 (KDR)	278 (61.8)	172 (38.2)	289 (64.2)	161 (35.8)	0.45
rs2305948 (KDR)	389 (86.4)	61 (13.6)	381 (84.7)	69 (15.3)	0.45
rs13109660 (KDR)	303 (67.3)	147 (32.7)	310 (68.9)	140 (31.1)	0.62
rs7671745 (KDR)	294 (65.3)	156 (34.7)	291 (64.7)	159 (35.3)	0.83
rs3124591 (NOTCH1)	402 (89.3)	48 (10.7)	400 (88.9)	50 (11.1)	0.83
rs9818496 (PDCD10)	391 (86.9)	59 (13.1)	394 (87.6)	56 (12.4)	0.77
rs3804610 (PDCD10)	392 (87.1)	58 (12.9)	396 (88.0)	54 (12.0)	0.69
rs6784267 (PDCD10)	286 (63.6)	164 (36.4)	286 (63.6)	164 (36.4)	1.00
rs833061 (VEGFA)	327 (72.7)	123 (27.3)	347 (77.1)	103 (22.9)	0.12
rs10434 (VEGFA)	361 (80.2)	89 (19.8)	345 (76.7)	105 (23.3)	0.20
rs833069 (VEGFA)	277 (61.6)	173 (38.4)	252 (56.0)	198 (44.0)	0.09
rs3025010 (VEGFA)	317 (70.4)	133 (29.6)	347 (77.1)	103 (22.9)	0.02^*∗*^
rs3025053 (VEGFA)	389 (86.4)	61 (13.6)	381 (84.7)	69 (15.3)	0.45
rs699947 (VEGFA)	327 (72.7)	123 (27.3)	333 (74.0)	117 (26.0)	0.65
rs2146323 (VEGFA)	337 (74.9)	113 (25.1)	351 (78.0)	99 (22.0)	0.27
rs3025035 (VEGFA)	376 (83.6)	74 (16.4)	373 (82.9)	77 (17.1)	0.79
rs3025030 (VEGFA)	373 (82.9)	77 (17.1)	366 (81.3)	84 (18.6)	0.54

Ref.: reference allele; alt.: altered (minor) allele. ^*∗*^*P* values have significance.

**Table 4:** The association between SNPs and hypertension.

Variables	Unadjusted^1^		Adjusted^2^	
	OR (95% CI)	*P* value	OR (95% CI)	*P* value
Additive model				
rs833061				
TT	Reference		Reference	
CT	1.114 (0.739, 1.680)	0.606	0.983 (0.423, 2.287)	0.969
CC	0.400 (0.184, 0.870)	0.021^*∗*^	0.219 (0.036, 1.342)	0.101
rs3025010				
TT	Reference		Reference	
CT	0.850 (0.563, 1.284)	0.441	0.476 (0.210, 1.079)	0.075
CC	0.392 (0.179, 0.858)	0.019^*∗*^	0.103 (0.019, 0.551)	0.008^*∗*^

Dominant model				
rs833061				
TT	Reference		Reference	
CT + CC	0.898 (0.620, 1.302)	0.571	1.458 (0.749, 2.840)	0.267
rs3025010				
TT	Reference		Reference	
CT + CC	0.730 (0.499, 1.068)	0.105	0.354 (0.147, 0.854)	0.021^*∗*^

Recessive model				
rs833061				
TT + CT	Reference		Reference	
CC	0.391 (0.181, 0.846)	0.017^*∗*^	0.114 (0.012, 1.073)	0.058
rs3025010				
TT + CT	Reference		Reference	
CC	0.409 (0.188, 0.888)	0.024^*∗*^	0.240 (0.042, 1.380)	0.110

^1^OR and *P* values: unadjusted values in univariate analysis. ^2^OR and *P* values: multivariate analysis. We adjusted age, gender, BMI, smoking, drinking, and physical activity for both rs833061 and rs3025010 in all three models. Besides, in the additive model, rs833069, rs3025010, rs2146323, rs3212278, rs2305948, rs7667298, rs7671745, and rs3025053 were adjusted for rs833061, and rs2305948, rs3212278, rs7671745, rs833069, and rs2146323 were adjusted for rs3025010; in the dominant model, rs3025010 and rs3212278 were adjusted for rs833061, and rs833061 and rs2146323 were adjusted for rs3025010; and in the recessive model, rs833069, rs3025010, rs2146323, rs7667298, and rs10434 were adjusted for rs833061, and rs833061, rs2146323, rs7667298, and rs10434 were adjusted for rs3025010. ^*∗*^*P* values have significance.

**Table 5:** The interaction between rs3025010 and SNPs in the *CCM3* gene.

Variables	Unadjusted^1^		Adjusted^2^	
	OR (95% CI)	*P* value	OR (95% CI)	*P* value
Additive model				
rs3025010 (TT)^*∗*^rs6784267 (CC)	Reference			
rs3025010 (CT)^*∗*^rs6784267 (CT)	0.699 (0.416, 1.172)	0.174	0.642 (0.355, 1.160)	0.142
rs3025010 (CC)^*∗*^rs6784267 (CT)	0.162 (0.036, 0.725)	0.017^*∗*^	0.102 (0.018, 0.574)	0.010^*∗*^
rs3025010 (CT)^*∗*^rs6784267 (TT)	1.111 (0.527, 2.342)	0.782	1.086 (0.466, 2.530)	0.849
rs3025010 (CC)^*∗*^rs6784267 (TT)	0.667 (0.111, 3.990)	0.657	0.453 (0.053, 3.843)	0.468
Dominant model				
rs3025010 (TT)*∗*rs6784267 (CC)	Reference			
rs3025010 (CT + CC) ^*∗*^				
rs6784267 (CT + TT)	0.678 (0.454, 1.013)	0.058^*∗*^	0.574 (0.338, 0.973)	0.039^*∗*^
Recessive model				
rs3025010 (CT + TT) ^*∗*^				
rs6784267 (CT + CC)	Reference			
rs3025010 (CC) ^*∗*^				
rs6784267 (TT)	0.667 (0.111, 3.990)	0.657	1.401 (0.167, 11.763)	0.756

^1^OR and *P* values: unadjusted values in univariate analysis. ^2^OR and *P* values: multivariate analysis. We adjusted age, gender, BMI, smoking, drinking, and physical activity in all three models. Besides, rs833069, rs2146323, rs3212278, rs2305948, and rs7671745 were adjusted in the additive model; rs833061 and rs2146323 were adjusted in the dominant model; and rs833061, rs2146323, rs7667298, and rs10434 were adjusted in the recessive model. ^*∗*^*P* values have significance.

## Data Availability

The data used to support the findings of this study are available from the corresponding author upon request.

## Supplementary Materials

Supplementary Materials Table 1 shows detailed SNP information. Figure 1 and Table 2 show the analysis results of propensity score matching. Doc 1 shows the output results of power tests. Table 3 shows the quality assessment result of genotyping.
