# Supplementary material for: Association between Single Nucleotide Polymorphisms in Cardiovascular Developmental Critical Genes and Hypertension: A Propensity Score Matching Analysis
Source: Int J Hypertens. 2020 Mar 19;2020:9185697. doi: 10.1155/2020/9185697 (PMC7106934; doi:10.1155/2020/9185697)
Supplement: Supplementary Materials — Table 1 shows detailed SNP information. Figure 1 and Table 2 show the analysis results of propensity score matching. Doc 1 shows the output results of power tests. Table 3 shows the quality assessment result of genotyping. [file 9185697.f1.zip › 9185697.f1/Supplemental Table 1.docx]

**Supplemental Table 1** The information of SNPs

| SNP | chr | Chr. Position | Gene | Ref mRNA | SNP Property | Alleles | Minor allele |
| --- | --- | --- | --- | --- | --- | --- | --- |
| rs2277538 | 15 | 41227050 | DLL4 | NM_019074.3 | intron7 | C/T | T |
| rs3212278 | 15 | 41224124 | DLL4 | NM_019074.3 | intron4 | G/A | G |
| rs55805015 | 15 | 41230575 | DLL4 | NM_019074.3 | 3'-UTR_exon11 | G/T | T |
| rs13109660 | 4 | 55970680 | KDR | NM_002253.2 | intron13 | G/A | A |
| rs2305948 | 4 | 55979558 | KDR | NM_002253.2 | nonsynon_exon7 | C/T | T |
| rs7667298 | 4 | 55991731 | KDR | NM_002253.2 | 5'-UTR_exon1 | C/T | T |
| rs7671745 | 4 | 55956836 | KDR | NM_002253.2 | intron22 | G/A | A |
| rs3124591 | 9 | 139390397 | NOTCH1 | NM_017617.3 | 3'-UTR_exon34 | C/T | C |
| rs73668310 | 9 | 139390517 | NOTCH1 | NM_017617.3 | 3'-UTR_exon34 | C/T | T |
| rs3804610 | 3 | 167413168 | PDCD10 | NM_007217.3 | intron6 | C/T | C |
| rs6784267 | 3 | 167426660 | PDCD10 | NM_007217.3 | intron3 | C/T | T |
| rs9818496 | 3 | 167450310 | PDCD10 | NM_007217.3 | intron2 | C/T | T |
| rs10434 | 6 | 43753212 | VEGFA | NM_003376.5 | 3'-UTR_exon8 | G/A | A |
| rs2146323 | 6 | 43745095 | VEGFA | NM_003376.5 | intron2 | C/A | A |
| rs3025010 | 6 | 43747577 | VEGFA | NM_003376.5 | intron5 | C/T | C |
| rs3025030 | 6 | 43750587 | VEGFA | NM_003376.5 | intron7 | G/C | C |
| rs3025035 | 6 | 43751359 | VEGFA | NM_003376.5 | intron7 | C/T | T |
| rs3025053 | 6 | 43753325 | VEGFA | NM_003376.5 | 3'-UTR_exon8 | G/A | A |
| rs699947 | 6 | 43736389 | VEGFA | NM_003376.5 | 5'-flanking | C/A | A |
| rs833061 | 6 | 43737486 | VEGFA | NM_003376.5 | 5'-flanking | C/T | C |
| rs833069 | 6 | 43742579 | VEGFA | NM_003376.5 | intron2 | C/T | C |
