# Supplementary material for: Association between Single Nucleotide Polymorphisms in Cardiovascular Developmental Critical Genes and Hypertension: A Propensity Score Matching Analysis
Source: Int J Hypertens. 2020 Mar 19;2020:9185697. doi: 10.1155/2020/9185697 (PMC7106934; doi:10.1155/2020/9185697)
Supplement: Supplementary Materials — Table 1 shows detailed SNP information. Figure 1 and Table 2 show the analysis results of propensity score matching. Doc 1 shows the output results of power tests. Table 3 shows the quality assessment result of genotyping. [file 9185697.f1.zip › 9185697.f1/Supplemental Table 3.docx]

**Supplemental Table 3** The quality assessment of genotyping

| **SNPs** | **Call Rate(%)** | **HWE (*P*)** | **MAF（observed in this study）** | | **MAF(HapMap-HCB)** | |
| --- | --- | --- | --- | --- | --- | --- |
| rs10434 | 98.19 | 0.9886 | A | 0.212 | A | 0.202 |
| rs13109660 | 98.19 | 0.7167 | A | 0.324 | A | 0.31 |
| rs2146323 | 98.19 | 0.1185 | A | 0.228 | A | 0.145 |
| rs2277538 | 98.19 | 0.6619 | T | 0.092 | T | 0.08 |
| rs2305948 | 98.19 | 0.0177 | T | 0.142 | T | 0.146 |
| rs3025010 | 98.19 | 0.3362 | C | 0.255 | C | 0.248 |
| rs3025030 | 98.19 | 0.1279 | C | 0.176 | C | 0.175 |
| rs3025035 | 98.19 | 0.3504 | T | 0.167 | T | 0.179 |
| rs3025053 | 98.19 | 1 | A | 0.139 | A | 0.099 |
| rs3124591 | 98.19 | 0.5136 | C | 0.054 | C | 0.058 |
| rs3212278 | 98.19 | 0.5397 | G | 0.312 | G | 0.273 |
| rs3804610 | 98.19 | 0.0328**^*^** | C | 0.125 | C | 0.096 |
| rs6784267 | 98.19 | 0.3801 | T | 0.372 | T | 0.371 |
| rs699947 | 98.19 | 0.0639 | A | 0.244 | A | 0.244 |
| rs7667298 | 98.19 | 0.5665 | T | 0.354 | T | 0.284 |
| rs7671745 | 98.19 | 0.2061 | A | 0.354 | A | 0.35 |
| rs833061 | 98.19 | 0.0639 | C | 0.244 | C | 0.243 |
| rs833069 | 98.19 | 0.5904 | C | 0.418 | C | 0.481 |
| rs9818496 | 98.19 | 0.0259 | T | 0.127 | T | 0.099 |
