# Supplementary figures and images for: Association between Single Nucleotide Polymorphisms in Cardiovascular Developmental Critical Genes and Hypertension: A Propensity Score Matching Analysis
Source: Int J Hypertens. 2020 Mar 19;2020:9185697. doi: 10.1155/2020/9185697 (PMC7106934; doi:10.1155/2020/9185697)

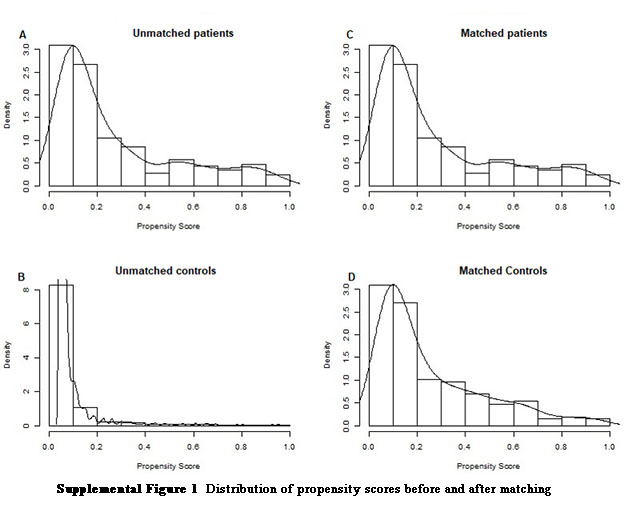

Supplement: Supplementary Materials — Table 1 shows detailed SNP information. Figure 1 and Table 2 show the analysis results of propensity score matching. Doc 1 shows the output results of power tests. Table 3 shows the quality assessment result of genotyping. [file 9185697.f1.zip › 9185697.f1/Supplemental Figure 1.jpg]
