# Supplementary material for: Association between Single Nucleotide Polymorphisms in Cardiovascular Developmental Critical Genes and Hypertension: A Propensity Score Matching Analysis
Source: Int J Hypertens. 2020 Mar 19;2020:9185697. doi: 10.1155/2020/9185697 (PMC7106934; doi:10.1155/2020/9185697)
Supplement: Supplementary Materials — Table 1 shows detailed SNP information. Figure 1 and Table 2 show the analysis results of propensity score matching. Doc 1 shows the output results of power tests. Table 3 shows the quality assessment result of genotyping. [file 9185697.f1.zip › 9185697.f1/Supplemental Doc1.doc]

PASS 15.0.5	2019/4/9 14:21:54	1

**Tests for Two Correlated Proportions in a Matched Case-Control Design**

**Numeric Results**

**Cases	Controls Per	Odds Ratio	Probability	Correlation**

**Power	(N)	Case (M)	(OR)	Exposed (P0)	(Phi)	Alpha	Beta**

0.58991	258	258	1.50	0.10000	0.20000	0.05000	0.41009

0.78689	258	258	1.50	0.20000	0.20000	0.05000	0.21311

0.86342	258	258	1.50	0.30000	0.20000	0.05000	0.13658

0.88968	258	258	1.50	0.40000	0.20000	0.05000	0.11032

0.88789	258	258	1.50	0.50000	0.20000	0.05000	0.11211

**References**

Dupont, William. 1988. 'Power Calculations for Matched Case-Control Studies', Biometrics, Volume 44, pages

1157-1168.

**Report Definitions**

Power is the probability of rejecting a false null hypothesis.

N is the size of the sample drawn from the treatment (case) group.

M is the number of matching control patients drawn for each case patient.

OR is the odds ratio of for subjects exposed to the risk factor.

P0 is the probability of exposure among sampled control patients.

Phi is the correlation of exposure between matched individuals.

Alpha is the probability of rejecting a true null hypothesis.

Beta is the probability of accepting a false null hypothesis.

**Summary Statements**

In a matched case-control study, the probability of exposure among sampled control patients is

0.10000 and the correlation coefficient for exposure between matched case and control patents

is 0.20000. A sample of 258 case patients is obtained. For each case patient, a matching sample

of 258 control patient(s) is also obtained. This sample of 66822 patients achieves 59% power to

detect an odds ratio of 1.50 versus the alternative of equal odds using a Chi-Square test with

a 0.05000 significance level.

**Dropout-Inflated Sample Size**

**Dropout-**

**Inflated	Expected**

**Enrollment	Number of**

**Sample Size	Sample Size	Dropouts**

**Dropout Rate	N	N'	D**

20%	258	323	65

**Definitions**

Dropout Rate (DR) is the percentage of subjects (or items) that are expected to be lost at random during the

course of the study and for whom no response data will be collected (i.e. will be treated as "missing").

N is the evaluable sample size at which power is computed (as entered by the user). If N subjects are

evaluated out of the N' subjects that are enrolled in the study, the design will achieve the stated power.

N' is the total number of subjects that should be enrolled in the study in order to end up with N evaluable

subjects, based on the assumed dropout rate. N' is calculated by inflating N using the formula N' = N / (1

- DR), with N' always rounded up. (See Julious, S.A. (2010) pages 52-53, or Chow, S.C., Shao, J., and Wang,

H. (2008) pages 39-40.)

D is the expected number of dropouts. D = N' - N.

PASS 15.0.5	2019/4/9 14:21:54	2

**Tests for Two Correlated Proportions in a Matched Case-Control Design**

**Chart Section**

PASS 15.0.5	2019/4/9 14:21:54	3

**Tests for Two Correlated Proportions in a Matched Case-Control Design**

**Procedure Input Settings**

**Autosaved Template File**

C:\Users\Lenovo\Documents\PASS 15\Procedure Templates\Autosave\Tests for Two Correlated Proportions in a Matched Case-Control Design - Autosaved 2019_4_9-14_21_54.t115

**Design Tab**

Solve For:	Power

Alpha:	0.05

N (Number of Case Patients):	258

M (Number of Controls per Case)	258

P0 (Probability that a Control is Exposed):	0.1 0.2 0.3 0.4 0.5

OR (Odds Ratio):	1.5

Phi (Correlation between Case and Control):	0.2
